# Association of CAD, a multifunctional protein involved in pyrimidine synthesis, with mLST8, a component of the mTOR complexes

**DOI:** 10.1186/1423-0127-20-24

**Published:** 2013-04-18

**Authors:** Akio Nakashima, Ippei Kawanishi, Sumiko Eguchi, Eugene Hsin Yu, Satoshi Eguchi, Noriko Oshiro, Ken-ichi Yoshino, Ushio Kikkawa, Kazuyoshi Yonezawa

**Affiliations:** 1 Biosignal Research Center, Kobe University, Kobe, 657-8501, Japan; 2 Current address: Exploratory Research Laboratories, Research Center, Ajinomoto Pharmaceuticals Co., Ltd., Kawasaki, 210-8681, Japan; 3 Current address: Yu’s Endocrine & Diabetes Clinic, Tainan, 700, Taiwan; 4 Current address: Department of Medical Biology, Akita University Graduate School of Medicine, Akita, 010-8543, Japan; 5 Current address: Department of Molecular Biology, Massachusetts General Hospital, Boston, MA, 02114, USA

**Keywords:** mLST8, CAD, mTOR, Amino acids, Pyrimidine synthesis

## Abstract

**Background:**

mTOR is a genetically conserved serine/threonine protein kinase, which controls cell growth, proliferation, and survival. A multifunctional protein CAD, catalyzing the initial three steps in *de novo* pyrimidine synthesis, is regulated by the phosphorylation reaction with different protein kinases, but the relationship with mTOR protein kinase has not been known.

**Results:**

CAD was recovered as a binding protein with mLST8, a component of the mTOR complexes, from HEK293 cells transfected with the FLAG-mLST8 vector. Association of these two proteins was confirmed by the co-immuoprecipitaiton followed by immunoblot analysis of transfected myc-CAD and FLAG-mLST8 as well as that of the endogenous proteins in the cells. Analysis using mutant constructs suggested that CAD has more than one region for the binding with mLST8, and that mLST8 recognizes CAD and mTOR in distinct ways. The CAD enzymatic activity decreased in the cells depleted of amino acids and serum, in which the mTOR activity is suppressed.

**Conclusion:**

The results obtained indicate that mLST8 bridges between CAD and mTOR, and plays a role in the signaling mechanism where CAD is regulated in the mTOR pathway through the association with mLST8.

## Background

Target of rapamycin (TOR) is a serine/threonine protein kinase genetically conserved among species from yeast to mammal, which belongs to the phosphatidylinositol 3-kinase-related kinase family among the kinome, and controls cell growth, proliferation, and survival. In mammal, mechanistic TOR (mTOR) exists as two independently regulated hetero-oligomeric complexes with common and respective partners, called mTOR complex 1 (mTORC1) and mTOR complex 2 (mTORC2), that are apparently sensitive and insensitive to rapamycin, respectively [1]. mTORC1, that contains raptor, mLST8 also named as GβL, and DEPTOR, directly phosphorylates the substrates such as S6 kinase, 4E-BP1, and PRAS40, and links signals including amino acids, growth factors, and energy to regulate various cellular functions such as protein synthesis, transcription, metabolism, and autophagy. Rheb, a small GTPase, activates mTORC1 as the GTP-bound form, and tuberous sclerosis complex (TSC) gene products, TSC1 and TSC2, form a dimer to function as a GTPase activating protein toward Rheb and thereby negatively regulate mTORC1. mTORC2, on the other hand, has rictor, mLST8, mSin1, DEPTOR, and Protor, and mediates growth factor signals but not those of nutrients to catalyze the phosphorylation of some AGC protein kinases such as Akt, SGK, and PKCα [2,3].

mLST8 is a 36-kDa protein having seven WD40 repeats, and is a common partner of mTORC1 and mTORC2 [4,5]. mLST8 appears to bind to the catalytic domain of mTOR [6], and the overexpression and knockdown of mLST8 suggested its positive function in mTORC1. The following studies using mice lacking mLST8 showed that this protein is required for assembly of mTOR and rictor, but not for the mTOR-raptor interaction, and that mLST8 has a role for mTORC2 rather than for mTORC1 [7]. The precise role of mLST8, thus, still remains unclear especially in mTORC1.

In this study, we isolated the binding proteins to mLST8 by analyzing the proteins that co-immunoprecipitated with FLAG epitope-tagged mLST8 from the transfected HEK293 cells, and identified one of them as CAD, a multifunctional protein with a deduced molecular mass of 243 kDa composed of the distinct regions in a single polypeptide: carbamoyl phosphate synthetase II (CPSase), aspartate transcarbamoylase (ATCase), and dihydroorotase (DHOase), which catalyze each of the initial three steps in *de novo* pyrimidine synthesis [8,9]. CPSase is the first and rate-limiting step for the nucleotide synthesis and allosterically activated and inhibited by phosphoribosyl 5’-pyrophosphate and uridine nucleotides, respectively. Moreover, CAD is regulated by the phosphorylation reaction with different protein kinases such as MAP kinase [10], PKA [11], and PKC [12]. Very recently, CAD has been reported to be phosphorylated by S6 kinase in the downstream of mTORC1 [13,14].

Here, we describe the association of CAD with mLST8, which provides a physical environment where CAD is regulated by the protein phosphorylation reaction in the mTOR signaling pathway, and an evidence that the CAD enzymatic activity is controlled in the mTOR-signaling pathway.

## Methods

### cDNAs

The FLAG-tagged expression vectors of the wild type mLST8 (FLAG-mLST8) and its mutants replacing Gly150 by Asp (G150D), Gly192 by Asp (G192D), and Phe320 by Ser (F320S) constructed in pCMV5 were kindly provided by Dr. Joseph Avruch (Massachusetts General Hospital, USA). The mLST8 mutant replacing Ala182 by Asp (A182D) was generated using a QuikChange site-directed mutagenesis kit (Stratagene). The expression vector of HA-tagged mTOR was constructed as described previously [15]. The cDNA encoding CAD was cloned by the successive polymerase chain reactions using mouse brain cDNAs (Quick-Clone, Clontech) as template. The primers were designed to amplify CAD in three portions according to the DNA sequence in the database (accession no. NM_023525), and the products were assembled into pcDNA3 with myc-epitope tag. The deletion mutants of CAD, GLN/CPS (amino acids 1–1456), GLN/CPS’ (amino acids 1–1461), DHO/ATC (amino acids 1457–2225), DHO/ATC’ (amino acids 1462–2225), GLN (amino acids 1–373), CPS-A (amino acids 391–939), CPS-B (amino acids 929–1461), DHO (amino acids 1457–1788), and ATC (amino acids 1911–2225) were generated in the pcDNA3-myc vector.

### Antibodies

The anti-FLAG (M2) and anti-myc (9E10) antibodies were purchased from Sigma, and the anti-HA antibodies (12CA5 and 3F10) were from Roche. The polyclonal antibody against mLST8 was generated as described [16]. The rabbit polyclonal anti-peptide antibody recognizing CAD was produced by the antibody service of Immuno-Biological Laboratories against the synthetic peptide EVDSDPRAAYFRQAENG (amino acids 2194–2210). Normal rabbit and mouse globulin were obtained from Santa Cruz Biotechnology. The horseradish peroxidase (HRP)-conjugated anti-mouse and anti-rabbit antibodies were obtained from Jackson ImmunoResearch Laboratories and Bio-Rad, respectively.

### Cell culture and transfection

HEK293 cells were maintained in Dulbecco’s modified Eagle’s medium (DMEM) (Sigma) containing 10% fetal bovine serum (FBS) (Gibco BRL) at 37°C in a 5% CO_2_ incubator. The cells were transfected with expression vectors by lipofection using lipofectamine (Invitrogen) according to the manufacturer’s protocol. For starvation of the cells, they were first incubated in DMEM without FBS for 16 h, and further incubated for 2 h with different culture media [17].

### Immunoprecipitation

The following procedures were carried out at 0-4°C. The cells were washed with ice-cold with Dulbecco’s phosphate-buffered saline, and lysed with Buffer A (20 mM Tris–HCl at pH 7.5, 120 mM NaCl, 1 mM EDTA, 5 mM EGTA, 20 mM β-glycerophosphate, 0.3% CHAPS, 1 mM phenylmethylsulfonyl fluoride, 2 μg/ml aprotinin, 2 μg/ml leupeptin, and 1 mM dithiothreitol). The supernatant was recovered by centrifugation at 15,000 × g for 25 min, and was incubated for 2 h with Protein G-Sepharose (GE Healthcare) coupled with each antibody, and the immunoprecipitate was washed three times with Buffer A.

### Mass spectrometry

The immunoprecipitate was obtained by the anti-FLAG antibody from the HEK293 cells transfected with FLAG-mLST8. The resin was eluted with Buffer A containing 200 μg/ml FLAG peptide (Sigma), and the proteins were separated by SDS-PAGE and visualized by silver staining. Each protein band was recovered and mass spectrometric analysis was carried out essentially as described [17].

### Immunoblot

The cell extracts and immunoprecipitates were separated by SDS-PAGE, and the proteins were transferred to a polyvinylidene difluoride membrane and subjected to immunoblotting using each primary antibody. After incubation with the HRP-conjugated secondary antibodies, detection of the proteins was carried out by the chemiluminescence reaction.

### mTOR kinase assay

The mTOR kinase assay was performed as previously described [16,17]. Briefly, myc-CAD was immunoprecipitated from the transfected HEK293 cells and eluted from the resign with 300 μg/ml myc peptide (Fujiya, Japan). The immunoprecipitates obtained by the anti-HA antibody from the HEK293 cells transfected with either HA-mTOR or the empty vector were incubated with purified CAD in the reaction mixture containing 10 mM HEPES at pH7.4, 50 mM β-glycerophosphate, 50 mM NaCl, 10 mM MnCl_2_, 100 μM ATP, and 370 kBq of [γ-^32^P]ATP. After the incubation for 30 min at 30°C, the samples were separated by SDS-PAGE, transferred onto a polyvinylidene difluoride membrane, and analyzed by autoradiography.

### CPSase activity assay

The CPSase activity of the immunoprecipitated myc-CAD was measured essentially as described [18,19]. Briefly, HEK293 cells were transfected with the myc-CAD expression vector, cultured under the different conditions, and lysed with Buffer A containing 20 mM NaF, 10 mM *p*-nitrophenyl phosphoric acid, 30% dimethylsulfoxide, and 5% glycerol. The immunoprecipitate with the anti-myc antibody was washed with 100 mM Tris–HCl at pH 8.0 containing 100 mM KCl, 7.5% dimethylsulfoxide, and 2.5% glycerol, and then incubated for 20 min at 37°C with 500 μl of the reaction mixture containing 100 mM Tris–HCl at pH 8.0, 100 mM KCl, 7.5% dimethylsulfoxide, 2.5% glycerol, 1 mM dithiothreitol, 3.5 mM glutamine, 20.2 mM aspartate, 1.5 mM ATP, 200 mM phosphoribosyl 5’-pyrophosphate, 3.5 mM MgCl_2_, and 5 mM [^14^C]NaHCO_3_ (118 MBq/mmol). The reaction was stopped by the addition of 500 μl of 40% trichloroacetic acid, and the tubes were heated at 95°C for 3 h. The incorporation of the ^14^C radioactivity into the acid stable materials was then measured by liquid scintillation counting, and the relative activity in each immunoprecipitate was normalized to the amount of immunoprecipitated myc-CAD detected by immunoblot.

### Statistical analysis

The result is expressed as the mean ± SD of data obtained from quadruplicate experiments. Statistical analysis was performed using a paired Student t-test, and a *P* value less than 0.01 was considered statistically significant.

## Results

### Identification of CAD as an mLST8-binding protein

**Figure 1:**
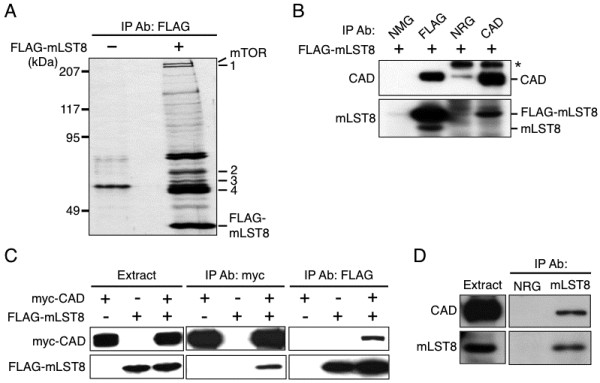
**Identification of CAD as an mLST8-binding protein.** (**A**) Co-purification of CAD with FLAG-mLST8. The extract from HEK293 cells transfected with or without FLAG-mLST8 vector was subjected to immunoprecipitation with the anti-FLAG antibody, and the proteins eluted from resin with the FLAG peptide were visualized by silver staining after SDS-PAGE. FLAG-mLST8, mTOR, and the proteins recovered from the immunoprecipitate (No.1 to 4) are shown. The positions of the size standards are indicated in kDa. (**B**) Association of CAD with overexpressed mLST8. The extract from HEK293 cells transfected with the FLAG-mLST8 vector was subjected to immunoprecipitation with either the anti-CAD or anti-FLAG antibody, and the immunoprecipitated proteins were analyzed by immunoblot with the anti-CAD and anti-mLT8 antibodies. NRG (normal rabbit globulin) and NMG (normal mouse globulin) were employed as negative controls. *: a non-specific protein. (**C**) Association between myc-CAD and FLAG-mLST8. The extract from HEK293 cells transfected with myc-CAD and FLAG-mLST8 vectors was subjected to immunoprecipitation with either the anti-myc or anti-FLAG antibody, and the immunoprecipitated proteins were analyzed by immunoblot with antibodies. (**D**) Association between the endogenous CAD and mLST8. The HEK293 cell extract without transfection and the immunoprecipitate of the extract with the anti-mLST8 antibody were analyzed by immunoblot with the anti-CAD and anti-mLT8 antibodies. NRG was employed as a negative control.

Proteins associating with mLST8 were searched by comparing the proteins retrieved in the anti-FLAG immunoprecipitate prepared from HEK293 cells transfected with the FLAG-mLST8 vector or an empty vector. The co-immunoprecipitation with FLAG-mLST8 followed by elution with the FLAG peptide recovered several proteins as visualized by silver staining after SDS-PAGE (Figure 1A). Mass spectrometric analysis revealed that the cellular proteins included FLAG-mLST8 itself and mTOR, and the proteins indicated as numbers 1 to 4 of approximate molecular mass from 240 to 60 kDa in Figure 1A were CAD, T-complex protein α and ε, T-complex protein η and θ, and tubulin β5, respectively (data not shown). We focused our attention on the protein number 1, CAD, because this protein is a rate-limiting enzyme for the pyrimidine synthesis and is essential for the regulation of cell growth [8,9]. Immunoblot analysis using the anti-CAD antibody confirmed that CAD is co-immunoprecipitated with FLAG-mLST8 by the anti-FLAG antibody recovered from HEK293 cells transfected with the FLAG-mLST8 vector (Figure 1B). The anti-FLAG immunoprecipitate also contained not only FLAG-mLST8 but the endogenous mLST8, suggesting that CAD forms a multimeric complex with mLST8. On the other hand, the anti-CAD immunoprecipitate contained FLAG-mLST8 as expected, but the endogenous mLST8 was not found. It seems that the anti-mLST8 antibody is not sensitive enough to detect mLST8 co-immunoprecipitated with the endogenously expressed CAD. The association of CAD and mLST8 was further examined by overexpressing both proteins with different epitope tags in HEK293 cells: analysis showed that FLAG-mLST8 and myc-CAD were detected in the anti-myc and anti-FLAG immunoprecipitates, respectively (Figure 1C). CAD endogenously expressed was also found in the anti-mLST8 immunoprecipitate obtained from the untransfected cells (Figure 1D).

### The binding region of CAD with mLST8

**Figure 2:**
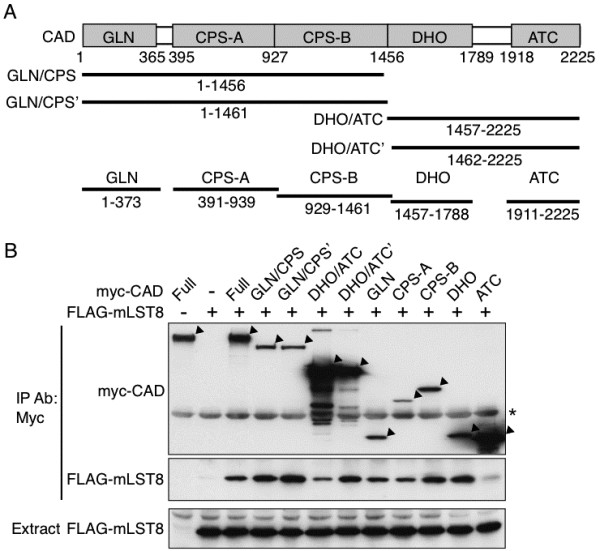
**The interaction region of CAD with mLST8.** (**A**) The schematic structure of CAD and the fragments employed. CAD and each fragment are shown with the amino acid numbers. (**B**) Association of mLST8 with the CAD fragments. HEK293 cells were transfected with the FLAG-mLST8 vector and Myc-tagged CAD expression vector of either the full length or each deletion mutant, and the cell extracts were subjected to immunoprecipitation with the anti-myc antibody. The extracts and immunoprecipitates were analyzed by immunoblot with the anti-myc or anti-FLAG antibody. The positions of CAD and its fragments are indicated by arrowheads. *: a non-specific protein.

CAD includes the distinct regions, CPSase, ATCase, and DHOase, which catalyze each of the initial three steps in *de novo* pyrimidine synthesis, and is composed of five domains of GLN (glutamine amidotransferase), CPS-A, CPS-B, DHO, and ATC [8]. A series of deletion mutants of CAD were thus generated to examine the binding region of mLST8 to CAD (Figure 2A, B). CAD was separated into the amino- and calboxyl-terminal halves of GLN/CPS (amino acids 1–1456) and DHO/ATC (amino acids 1457–2225), and alternatively into GLN/CPS’ (amino acids 1–1461) and DHO/ATC’ (amino acids 1462–2225) to avoid damage of the domains by the artificial cleavage. The binding of mLST8 was, rather unexpectedly, found in the immunoprecipitate of both amino- and calboxyl-terminal fragments. Therefore, CAD was further divided into each domain such as GLN (amino acids 1–373), CPS-A (amino acids 391–939), CPS-B (amino acids 929–1461), DHO (amino acids 1457–1788), and ATC (amino acids 1911–2225). Each domain, except for the ATC domain, showed a significant binding activity. These results suggested that mLST8 binds with CAD at more than one region rather than a single recognition site in agreement with those in Figure 1B.

### The binding of mLST8 with CAD and mTOR

**Figure 3:**
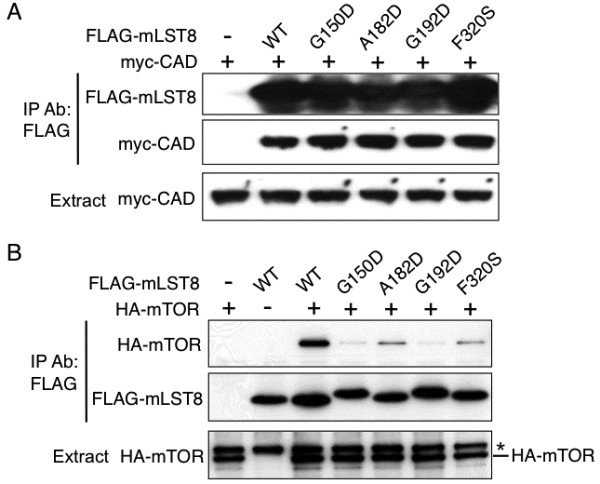
**Association of the mLST8 point mutants with CAD and mTOR.** (**A**) Association of the mLST8 point mutants with CAD. HEK293 cells were transfected with the myc-CAD vector and the FLAG-tagged expression vector of either the wild type or each point mutant of mLST8 as indicated. The extracts and immunoprecipitates with the anti-FLAG antibody were analyzed by immunoblot with the anti-FLAG and anti-myc antibodies. (**B**) Association of the mLST8 point mutants with mTOR. HEK293 cells were transfected with the HA-mTOR vector and the FLAG-tagged expression vector of either the wild type or each point mutant of mLST8 as indicated. The immunoprecipitates with the anti-FLAG antibody were analyzed by immunoblot with the anti-HA and anti-FLAG antibodies. *: a non-specific protein.

mLST8 is a component of the mTOR complexes, and thus the binding between mLST8 and CAD (Figure 3A) was compared with that between mLST8 and mTOR (Figure 3B) using mLST8 point mutants. mLST8 has some amino acid residues critical for the interaction with mTOR such as Gly150, Gly192, and Phe320 [6] as well as Ala182 (unpublished observation). The mutation in these residues eliminated or heavily reduced the binding with mTOR (Figure 3B), but these mutants associated with CAD as the wild type protein (Figure 3A). Namely, mLST8 associates with CAD in manner distinct from that with mTOR.

### The measurement of the CPSase activity

**Figure 4:**
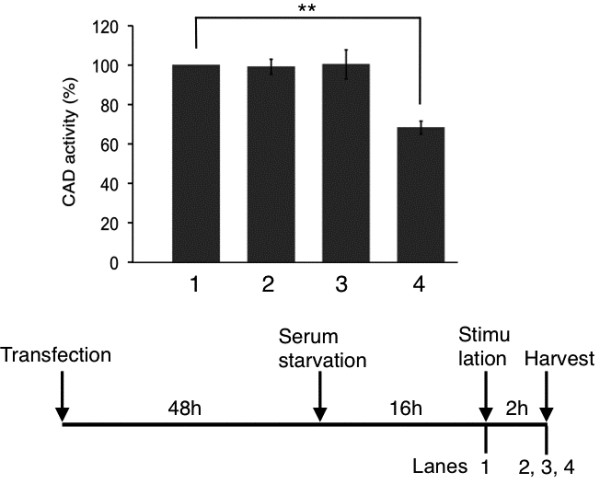
**The CPSase activity in the amino acid-depleted cells.** HEK293 cells transfected with the myc-CAD vector were cultured in the absence of serum for 16 h, and further incubated under different conditions for 2 h. Cells were harvested as shown in the bottom of the figure. 1: after serum starvation for 16 h, 2: after further incubation with serum, 3: after further incubation without serum, 4: after further incubation without serum and amino acids. The immunoprecipitates with the anti-myc antibody from the cells after each treatment were subjected to the CPSase assay. The relative CPSase activity normalized to the amount of immunoprecipitated myc-CAD are employing the activity of the serum-starved cells as 100%. **, *P* < 0.01.

The binding of CAD with mLST8 suggests that mTOR controls not only protein synthesis but also that of the pyrimidine nucleotides. It seemed possible that mTOR phosphorylates CAD to regulate its enzymatic activity, and thus *in vitro* incubation of myc-CAD with HA-mTOR was carried out in the presence of [γ-^32^P]ATP (Additional file 1). The phosphorylation of myc-CAD was, however, not detected under the conditions that the autophosphorylation of HA-mTOR [16] is clearly observed. Then, the CPSase activity was measured as a representative of this multienzymatic protein. HEK293 cells transfected with the myc-CAD vector were cultured in the absence of serum, incubated further under the different culture conditions, and then the immunoprecipitates with the anti-myc antibody were prepared from the cells and subjected to the assay of the CPSase activity (Figure 4). The CAD protein recovered from the cells cultured without amino acids, in which the mTORC1 activity is suppressed [20], showed the CPSase activity significantly lower than that obtained from the cells incubated with amino acids. The stimulation of the cells with serum, however, did not enhance the CPSase activity.

## Discussion

In this study, we identified CAD as a binding protein of mLST8. CAD was also detected in the screening of the binding proteins of FLAG-raptor, but the association was weaker than that with FLAG-mLST8 (data not shown). It is thus reasonable to assume that CAD associates with mTORC1 including raptor through mLST8. As mLST8 and raptor, however, both contain WD-40 repeats known to act as a site for protein-protein interaction [21], it is difficult to exclude the possibility that these two proteins recognize CAD, independently. Concerning the interaction between CAD and mLST8, the results obtained suggest that CAD has more than one region for the recognition of mLST8. It might be possible that the CAD fragments interact with endogenously expressed CAD and the CAD complexes thus generated associate with mLST8, because CAD forms oligomers [8,9]. On the other hand, mLST8 point mutants having little binding activity with mTOR still associated with CAD. Therefore, mLST8 may bridge between mTOR and CAD, and CAD binds to mLST8 in multiple ways.

We next examined the possibility that CAD is a substrate for mTORC1, because CAD is known to be phosphorylated by some protein kinases [10-12]. The *in vitro* phosphorylation of CAD was, however, not detected after the incubation with the mTOR immunoprecipitated from the transfected cells. Very recently, Robitaille *et al.*[13] have carried out the quantitative phosphoproteomics of the mTOR targets in the cells lacking raptor, and concluded that mTOR1 indirectly phosphorylates and regulates CAD: CAD is phosphorylated by S6 kinase at Ser1859 and thereby pyrimidine synthesis is stimulated. Ben-Sahra *et al.*[14], on the other hand, have identified Ser1859 and Ser1900 as the phosphorylation sites in CAD. They indicate that the modification of Ser1900 is stable but that of Ser1859 is stimulated by insulin in a manner sensitive to rapamycin, and propose that S6 kinase is responsible for the phosphorylation of Ser1859. These two studies indicate that CAD is controlled by the S6-kinase-mediated phosphorylation, but the practical interaction of CAD with mTOR1 has not been analyzed. Taken the available evidence together, it is reasonable to conclude that CAD associated with mTORC1 through mLST8 is not phosphorylated directly by mTOR, but is recognized by S6 kinase that is activated by mTORC1. It is interesting to examine the possibility that the binding proteins to mLST8 are phosphorylated by S6 kinase.

Ser1859 was identified as a phosphorylation site by S6 kinase [13,14], but this residue as well as Ser1406 have been shown to be the PKA sites in CAD [11]. CAD is, in addition, phosphorylated at Thr456 by MAP kinase [10] and at Ser1873 by PKC [12]. It is appropriate that CAD is phosphorylated in different signaling pathways by each upstream protein kinase, because the *deno nove* pyrimidine synthesis is the metabolic reactions critical for the cells. Moreover, CAD is regulated in coordinated manners through the phosphorylation by different protein kinases: MAP kinase and PKA phosphorylate CAD in a mutually antagonistic manner [22], and the PKC-dependent phosphorylation enhances the MAP kinase-mediated CAD activation [12]. It is thus important to analyze the regulation mechanisms of CAD from a wide point of view.

Concerning the *de novo* pyrimidine synthesis, Ben-Sahra *et al.*[14] have showed the regulation of the *de novo* pyrimidine synthesis through mTOR by comparing the metabolites in TSC2-deficient and control cells labeled with ^15^ N-glutamine. Robitaille *et al.*[13] have employed HeLa cells metabolically labeled with the ^15^ N-glutamine and reported the increase of dihydroorotate, orotate, and UTP after depletion and stimulation with both serum and amino acids. We revealed that the CPSase activity of the immunoprecipitated CAD recovered from the amino acid-depleted cells, in which the mTORC1 activity is suppressed, is decreased by the *in vitro* assay using [^14^C] NaHCO_3_, indicating the contribution of the mTOR complex in the regulation of the CAD activity. These results are compatible with those reported [13,14] and support the direct regulation of CAD by S6 kinase, but the CPSase activity, in this study, was not enhanced by serum stimulation. It is not clear why the CPSase activity was insensitive to serum stimulation. Further studies are necessary to clarify the regulation mechanisms of CAD through the phosphorylation reaction by the protein kinases.

CAD has been shown to bind with other proteins such as Rad9 [19] and androgen receptor [23]. Rad9 plays a role in the DNA damage checkpoint response as a damage sensor, and the binding of Rad9 to the CPSase domain stimulates the CPSase activity, suggesting the relationship between the DNA damage response and pyrimidine synthesis. Androgen receptor shows a facilitated nuclear localization and an enhanced transcriptional activity by the interaction with CAD, but the role of this nuclear receptor in nucleotide synthesis is not known. It is interesting to know the relationship between mTORC1 and these CAD binding proteins.

The evidences obtained indicate that mTORC1 is involved in the regulation of the pyrimidine nucleotide synthesis. The precise studies of mTORC1 and CAD will bring key observation to clarify the interplay between the synthesis of the proteins and nucleotides, which are critical for the proliferation of the cells.

## Conclusions

The results obtained indicate that mLST8 bridges between CAD and mTOR. CAD is not a direct phosphorylation substrate of mTOR, but the association of CAD with mLST8 provides a physical environment where CAD is regulated through the phosphorylation reaction in the mTORC1-signaling pathway.

## Competing interests

The authors declare no potential conflict of interests.

## Authors’ contributions

AN and IK carried out the major experiments. SuE, EHY, SaE, and KeY participated in the vector construction and mass spectrometric analysis. AN, NO, and UK participated in the analysis and interpretation of the data, and wrote the manuscript. KaY initiated this project and contributed to the experimental design. All authors read and approved the final manuscript.

## Supplementary Material

Additional file 1 **Incubation of myc-CAD with HA-mTOR in the kinase assay mixture.** The kinase reaction using [γ-^32^P]ATP was carried out in the presence of myc-CAD and HA-mTOR as indicated, and the samples were analyzed by autoradiography and immunoblotting after the separation by SDS-PAGE. The positions of myc-CAD and phosphorylated HA-mTOR are indicated in the autoradiograph. Click here for file
