# Supplementary figures and images for: Association of CAD, a multifunctional protein involved in pyrimidine synthesis, with mLST8, a component of the mTOR complexes
Source: J Biomed Sci. 2013 Apr 18;20(1):24. doi: 10.1186/1423-0127-20-24 (PMC3639846; doi:10.1186/1423-0127-20-24)

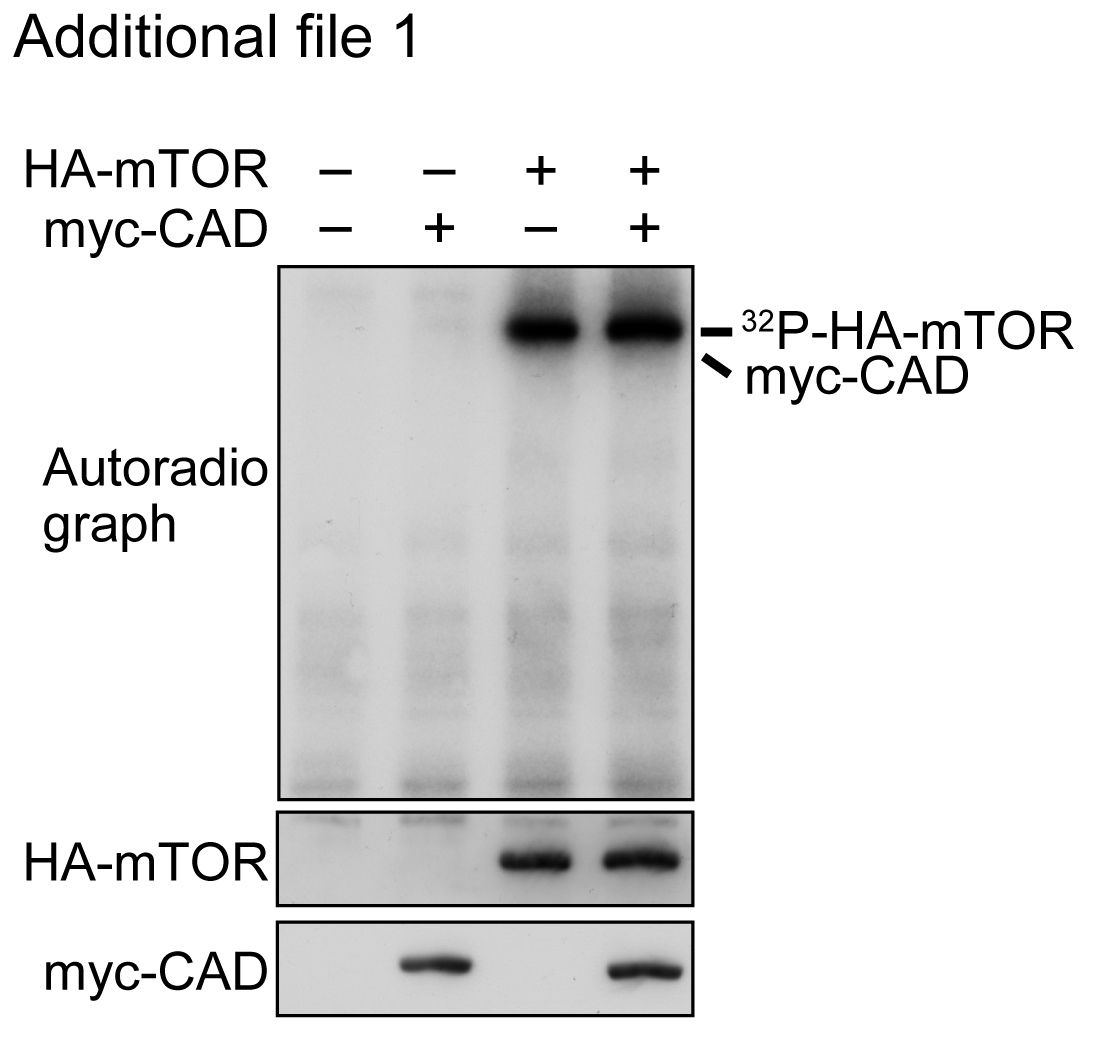

Supplement: Additional file 1 — Incubation of myc-CAD with HA-mTOR in the kinase assay mixture. The kinase reaction using [γ-32P]ATP was carried out in the presence of myc-CAD and HA-mTOR as indicated, and the samples were analyzed by autoradiography and immunoblotting after the separation by SDS-PAGE. The positions of myc-CAD and phosphorylated HA-mTOR are indicated in the autoradiograph. [file 1423-0127-20-24-S1.tiff]
